# Favorable subgingival plaque microbiome shifts are associated with clinical treatment for peri-implant diseases

**DOI:** 10.1038/s41522-024-00482-z

**Published:** 2024-02-19

**Authors:** Davide Bazzani, Vitor Heidrich, Paolo Manghi, Aitor Blanco-Miguez, Francesco Asnicar, Federica Armanini, Sara Cavaliere, Alberto Bertelle, Federico Dell’Acqua, Ester Dellasega, Romina Waldner, Daniela Vicentini, Mattia Bolzan, Cristiano Tomasi, Nicola Segata, Edoardo Pasolli, Paolo Ghensi

**Affiliations:** 1 PreBiomics S.r.l., Trento, Italy; 2 Department CIBIO, University of Trento, Trento, Italy; 3 Department of Agricultural Sciences, University of Naples Federico II, Portici, Italy; 4 Private Practice, Trentino-Alto Adige, Italy; 5 Department of Periodontology, Institute of Odontology, Sahlgrenska Academy, University of Gothenburg, Gothenburg, Sweden

**Keywords:** Plaque, Metagenomics

## Abstract

We performed a longitudinal shotgun metagenomic investigation of the plaque microbiome associated with peri-implant diseases in a cohort of 91 subjects with 320 quality-controlled metagenomes. Through recently improved taxonomic profiling methods, we identified the most discriminative species between healthy and diseased subjects at baseline, evaluated their change over time, and provided evidence that clinical treatment had a positive effect on plaque microbiome composition in patients affected by mucositis and peri-implantitis.

Millions of dental implants are placed every year, with about half of patients affected by important diseases such as mucositis (>50% of implant patients) and peri-implantitis (~20%)^1^. Several microorganisms have been suggested to play a role in the onset and progression of these clinical conditions^2,3^, and the comprehensive characterization of the oral and plaque microbiota through metagenomic sequencing could substantiate these links and enable new diagnostic and prognostic tools. Overcoming the limitations of existing studies that adopted low-resolution 16 S rRNA gene sequencing approaches^4–8^, we have recently performed the first metagenomic analysis of the microbiome in peri-implant diseases (*N* = 113 metagenomes from 72 individuals)^9^, identifying strong microbial signatures for mucositis and peri-implantitis that have been confirmed by other metagenomic investigations^10,11^. Such analyses have been focused on cross-sectional case–control settings with sampling of plaque microbiota before therapy. Despite some studies demonstrated that oral and plaque microbiomes can change in response to therapy and with respect to the level of success of the therapy^12–16^, no longitudinal studies have been conducted through metagenomics so far limiting our understanding of the microbiota dynamics associated with progression and treatment of oral diseases.

In this study, we performed a longitudinal pre/post-therapy metagenomic investigation of the subgingival plaque microbiome associated with mucositis and peri-implantitis. We extended our previously described cohort^9^ to 91 subjects and 320 quality-controlled metagenomes (for a total of 347 G bases of sequencing data) spanning healthy (*n* = 32), mucositis (*n* = 28), and peri-implantitis (*n* = 31) subjects (Supplementary Data S1 and Supplementary Data S2). Diseased subjects underwent surgical [mucositis: *n* = 2 ; peri-implantitis: *n* = 9] or nonsurgical treatments [mucositis: *n* = 26 (*n* = 2 mechanical instrumentation; *n* = 5 mechanical instrumentation + professional antimicrobial + home antimicrobial; *n* = 8 mechanical instrumentation + home antimicrobial; *n* = 11 home antimicrobial); peri-implantitis: *n* = 22 (*n* = 3 mechanical instrumentation; *n* = 8 mechanical instrumentation + professional antimicrobial + home antimicrobial; n = 4 mechanical instrumentation + home antimicrobial; *n* = 7 home antimicrobial)] decided by clinical practitioners, while healthy subjects did not receive any medications or therapies. Subjects were sampled at baseline (T0) and after intervention at 1 month (T1) and 6 months (T2) (“Methods”). As additional intra-subject controls, we sampled implants and/or teeth from the healthy sites that were contralateral to the diseased ones. Reference-based taxonomic profiles were generated using MetaPhlAn 4^17^ that delineates taxa at the resolution of species-level genome bins (SGBs) and is able to capture species that still miss cultivated representatives (“Methods”). In total, we detected 137 SGBs having an average relative abundance >0.1% in our set of metagenomes.

We first verified that the plaque microbiome was different among the three conditions at baseline (Fig. 1a; T0, PERMANOVA *P* < 0.001, “Methods“). Then, we expanded the analysis longitudinally to assess the effects of treatment on the microbiome composition. At T1, microbial composition of peri-implantitis samples changed significantly and shifted toward a healthier microbiome (Fig. 1a–c), consistently with a treatment-associated clinical improvement. A shift toward the configuration of the health plaque microbiome was further strengthened at 6 months (T2) after therapy administration (Fig. 1a–c), which suggested a long-term impact of the intervention on the microbiome composition. Nevertheless, the microbiome of two diseased implants bounced back into the disease-associated microbiome configuration at 6 months after initial favorable changes (Fig. 1a). Such observations were supported by temporal changes in probability density functions associated with healthy microbiome profiles at both taxonomic (Wilcoxon–Mann–Whitney *P* = 0.012 between T0 and T1; Wilcoxon–Mann–Whitney *P* = 2.9e-3 between T0 and T2; Fig. 1b) and functional levels (Wilcoxon–Mann–Whitney *P* = 0.045 between T0 and T1; Wilcoxon–Mann–Whitney *P* = 6.9e-3 between T0 and T2; Fig. 1c). Similar trends were observed when comparing mucositis with healthy samples. Specifically, the microbial composition for the diseased subjects shifted toward the healthy configuration after treatment with significant changes at T2 for both taxonomic (Wilcoxon–Mann–Whitney *P* = 0.019 between T1 and T2; Fig. 1b) and functional (Wilcoxon–Mann–Whitney *P* = 0.023 between T1 and T2; Fig. 1c) profiles. This microbiome shift toward a healthier state was significantly more evident among diseased individuals that showed clinical improvement of the peri-implant condition at T1 or T2 (Supplementary Fig. 1). Finally, when considering contralateral samples as controls, we did not get any statistically significant changes when comparing samples before and after treatment for the primary site (at both T1 and T2) independently from the disease status (Supplementary Fig. 2), which is in line with the site-specificity of the peri-implantitis microbiome.

**Fig. 1:**
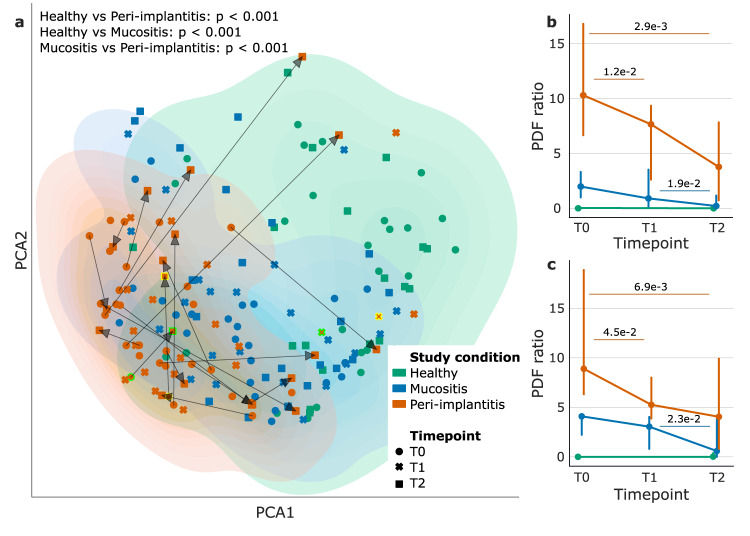
The plaque microbiome in peri-implantitis sites shifts toward a healthier microbiome after intervention. **a** Species-level microbiome composition was different at T0 among study conditions (*P* < 0.001, PERMANOVA). Arrows indicate trajectories of peri-implantitis samples from T0 to T2. Two patients that showed initial microbiome improvement at T1 and later bounced back at T2 are highlighted with a different color (light green or yellow). We assessed proximity to the healthy implant microbiome composition by considering probability density functions (PDFs). Such PDFs were computed on diseased samples and normalized on the healthy ones for both **b** taxonomic and **c** functional data (“Methods”; *P* values computed using Wilcoxon–Mann–Whitney test). Variations in such PDF ratios showed a post-treatment shift of the microbiome initially associated with disease to the configuration of the microbiome in healthy implants.

We then identified the species-level genome bins (SGBs) differentially abundant between healthy and peri-implantitis subjects at baseline by evaluating log odd ratios and the significance of the difference in the relative abundances (Fig. 2). Baseline SGB biomarkers of healthy implants tended to increase their abundance in peri-implantitis patients after their treatment at T2 (Fig. 2a), which further suggests the establishment of a healthier plaque microbiome composition. Most significant increases were observed for *Actinomyces oris*^18^ (*P* < 0.05) and *Corynebacterium matruchotii*^19^ (*P* < 0.05), with such variations also occurring in mucositis subjects. Both species belong to the Actinobacteria phylum, are primary colonizers of the subgingival microbiota, and play an important role in biofilm formation and stability^20^. On the other hand, some of the peri-implantitis biomarkers decreased in peri-implantitis patients after treatment (Fig. 2c). More specifically, the highest decreases in relative abundances between T0 and T2 were detected for *Porphyromonas gingivalis* (*P* < 0.05), *Fusobacterium nucleatum* SGB6013 (*P* < 0.05), and an uncharacterized species belonging to the *Anaerolineaceae* family (SGB17621; *P* < 0.05). While the role of the latter in peri-implantitis is unknown, the first two are classic periodontal pathogens that possibly must be cleared out from diseased sites for clinical improvement^20^. As a control, healthy patients showed minimal longitudinal changes in both sets of healthy and peri-implantitis SGB biomarkers, none of which were statistically significant (Fig. 2a, c). Contralateral sites showed some significant variations, but they were inconsistent and were also observed among healthy patients, suggestive of normal microbiome longitudinal variability independent from treatment (Fig. 2b, d). Finally, we extended this analysis strategy to evaluate changes in microbial functions in terms of gene family relative abundances. We similarly observed that functions associated with health increased in peri-implantitis patients after treatment (Supplementary Fig. 3a), while functions associated with peri-implantitis decreased (Supplementary Fig. 3b).

**Fig. 2:**
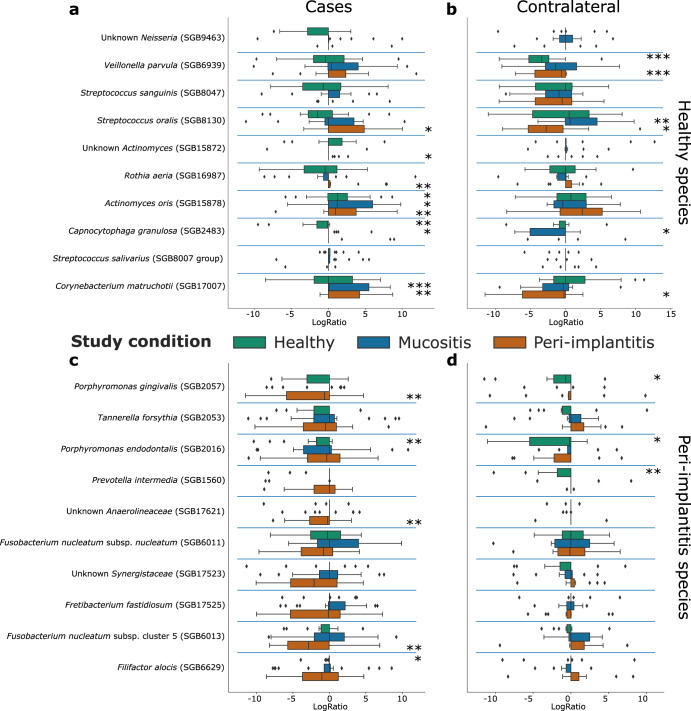
The plaque microbiome changed in peri-implantitis sites after the intervention. We report changes in relative abundances as the (base 2 logarithm) ratio of the abundances at T2 and T0. This logRatio is reported for (**a**, **c**) case and (**b**, **d**) contralateral samples and stratified by study condition. Contralateral samples were grouped according to the diagnosis of the respective case implant. We report the top-10 SGBs identified as the ones most enriched in (**a**, **b**) health and (**c**, **d**) peri-implantitis through LEfSe (“Methods”). *P* value: *<0.1; **<0.05; ***<0.01. Whiskers are ±SD.

Altogether, these results provide multiple evidence that the microbiota associated with peri-implant diseases can be reverted at least partially by effective clinical treatment to a composition compatible with the health status. Further investigations on a larger sample size may allow understanding whether diseased implants that do not respond both clinically and microbially to therapy have specific and identifiable microbiota configurations. These findings might also open up interesting perspectives for precision medicine approaches in the dental field, both for microbiota modulation through health-related microbial species and for the possibility of performing antibiotic and/or antiseptic topical therapies based on the microbiome composition of the specific diseased sites.

## Methods

### Subject recruitment

This study was approved by the ethics committee of the University of Trento (no. 2015-024) and was conducted in accordance with the guidelines of the World Medical Association Declaration of Helsinki. Both male and female patients, having dental implants and a regular maintenance of their dental implants were recruited from six private practices in the Province of Trento (Italy). The study protocol was explained to each subject and a signed informed consent was obtained. Inclusion criteria included: good general health as evidenced by the medical history; being at least 18 years old; not having fewer than 8 teeth; having at least one functioning oral implant restored with crowns or prostheses for at least 1 year. Exclusion criteria included: pregnancy or lactation; human immunodeficiency virus; use of immunosuppressant medications, bisphosphonates, or steroids; use of chlorhexidine mouthwash or gel during the previous 2 weeks; oral prophylactic procedures within the preceding 3 months; intake of systemic antibiotics or probiotics within the past 6 months.

Patients were divided into three groups based on the health status of the implant: (a) healthy (H, patients with at least one healthy implant and no implants with mucositis or peri-implantitis), (b) mucositis (M, patients with at least one implant with mucositis and no implants with peri-implantitis), and (c) peri-implantitis (P, patients with at least one implant with peri-implantitis). Clinical evaluation of inflammation and/or presence of suppuration (SUP), as well as radiographic marginal bone level, were used to include the patients in one of the three groups according to the criteria delineated by the Consensus Report on Peri-implant Diseases^21^. Soft tissue without signs of bleeding on probing (BOP) or SUP, and detectable bone loss classified implants as healthy. On the other hand, the presence of inflammation in at least one site and the absence of radiographic bone loss following functional load was associated with peri-implant mucositis. Peri-implantitis was classified based on the presence in at least one site of inflammation and a bone loss >2 mm since the prosthesis installation (i.e., at least 1 year after loading). Peri-implant mucositis and peri-implantitis patients were treated with different surgical and/or non surgical interventions decided by each clinical practitioner.

### Data collection and clinical examination

Six experienced clinical practitioners were asked to collect plaque samples following a shared protocol for the examination, collection, and measurement procedures. For calibration samples were collected from a limited number of volunteer subjects to minimize potential bias in sampling procedures between different clinicians. Meetings were held every 3 months to ensure consistency of sampling and avoid dentist-specific batch effects (see Supplementary Fig. 6 in Ghensi et al.^9^). Medical and dental history, as well as gender and age were recorded. A full-mouth clinical examination was performed in addition to a site-specific radiography examination, when required. Information about smoking and alcohol consumption, systemic health, and taken medications were retrieved. Clinical parameters included: implant or tooth; site of sampling; diagnosis of implant age in terms of time from installation; implant system used and nature of reconstruction (i.e., single, fixed or removable implant); type of implant retention (i.e., screw, cement, conometric); radiographic peri-implant bone loss; width of the keratinized mucosa; peri-implant probing depth (PPD); plaque index (PI); bleeding on probing (BOP); and SUP. The latter four parameters (i.e., PPD, PI, BOP, and SUP) were measured at the buccal, mesial, lingual, and distal sites of the experimental implant (healthy, mucositis, or peri-implantitis site) in addition to a healthy contralateral implant (if present) or tooth. PI, BOP, and SUP were recorded on a binary scale (i.e., presence/absence) for each surface and PPD was measured to the nearest millimeter on the scale. In the case of mucositis and peri-implantitis, any eventual subsequent therapy was noted. All patients were pseudo-anonymized in the clinic by assigning a unique patient ID. All downstream analyses were performed using the pseudo-anonymized IDs and metadata.

### Cohort and patient’s clinical characteristics

We enrolled for this study 102 patients (51 males, 51 females; mean age 62.54 ± 9.85 years) from three categories: healthy (H: 40), mucositis (M: 29), and peri-implantitis (P: 33). Each patient contributed with one implant in addition to the healthy contralateral site. Eleven patients were excluded due to failure in DNA extraction or library preparation for sequencing (healthy: 8, mucositis: 1, peri-implantitis: 2). The patients were followed for 6 months and sampled at 1 (Timepoint 1, T1) and 6 (Timepoint 2, T2) months (Supplementary Data S1).

Considering the 91 patients who contributed with quality-controlled metagenomes (see Sequence preprocessing and taxonomic/functional potential profiling), 38 had a history of periodontitis while 53 were periodontally healthy; 17 were current smokers, 28 were former smokers, and 46 never smokers. In total, 5 patients had diabetes and 11 experienced peri-implantitis in the past (past peri-implantitis information was unknown for 7 patients).

More specifically on the oral health status of each subject, 6 patients had ≤5 remaining teeth, 8 patients had between 5 and 10 remaining teeth (5 < x ≤ 10), 22 patients had between 10 and 20 remaining teeth (10 < x ≤ 20), 31 patients had between 20 and 25 remaining teeth (20 < x ≤ 25), 24 patients had more than 25 remaining teeth. 18 patients had one dental implant, 16 patients had two dental implants, 17 patients had three dental implants, 14 patients had four dental implants, 26 patients had fiver or more dental implants. 18 patients had a frequency of home oral care of once per day, 43 patients twice per day, 27 patients three times per day, 3 patients four or more times per day.

Study groups were compared for clinical characteristics as well as for demographic and anamnestic variables. No significant differences were found among these groups in terms of age (ANOVA *P* value = 0.9), gender (Pearson Chi-square *P* value = 0.2), history of periodontitis (Pearson Chi-square *P* value = 0.087), smoking habit (Pearson Chi-square *P* value = 0.14), diabetes (Pearson Chi-square *P* value = 0.38).

Extensive clinical data were registered both for experimental implants and contralateral implants/teeth (Supplementary Data S2).

When clinical parameters for the experimental implants were considered at the implant level, a significant difference was observed for PPD (ANOVA *P* value < 0.001), BOP (Pearson Chi-square *P* value < 0.001), SUP (Pearson Chi-square *P* value < 0.001), and bone loss (ANOVA *P* value < 0.001); all these parameters were significantly higher in peri-implantitis than in healthy conditions. PI was instead nonsignificant (Pearson Chi-square *P* value = 0.20) among groups.

Clinical improvement at T1 and T2 was evaluated by reclassifying the treated implant as healthy, mucositis, or peri-implantitis using the same criteria aforementioned. Initially diseased individuals reclassified as healthy or mucositis (in the case of individuals initially diagnosed as having peri-implantitis) were considered to have an “improved” clinical condition, while patients showing the same classification present at T0 were considered to have a “stable” clinical condition. Clinical improvement was observed for 13/40 subjects at T1 and 19/39 subjects at T2. Improvement data was not available for three subjects at T1 and 1 subject at T2 (Supplementary Data S2).

### Sample collection, DNA extraction, and Illumina shotgun sequencing

The sampling protocol followed in this study for the microbiome sample collection was based on the one we previously validated^9^. A single implant and a contralateral site (preferably an implant, otherwise a tooth) were sampled with two technical replicates from each selected patient. In the case of multiple implants with the same tested condition, a single one was randomly selected for sampling. To access submucosal and subgingival plaque samples, saliva was excluded from the selected sites using cotton rolls and, after drying with an air syringe, supramucosal and supragingival plaque were removed with sterile cotton pellets. The required plaque samples were collected from the deepest probing site with individual sterile titanium Gracey curettes. Any eventual bleeding was stopped before technical replicate sampling to avoid contamination. After the collection, samples were immediately placed in separate Eppendorf 1.5-mL microcentrifuge tubes (Eppendorf, Hamburg, Germany) containing sterile SCF-1 buffer solution (50 mM Tris-HCl, pH 7.5; 1 mM EDTA, pH 8.0; 0.5% Tween-20)^22^ and frozen at −80 °C for later analysis. Total genomic DNA was isolated using the Qiagen Power Soil Pro Kit (Qiagen, Hilden, Germany): an additional enzymatic disruption step for complete lysis of Gram-positive and Gram-negative species was performed, following the manufacturer’s protocol. Isolated DNA was stored at **−**20 °C. Laboratory control extractions were also performed on the prepared sample buffer to ascertain any potential contaminants. Each metagenome was first quantified, and when there was sufficient material (>1 ng), libraries were prepared using the Illumina DNA Prep Kit (Illumina Inc., San Diego, CA, USA) using the manufacturer’s protocol. Technical replicates were used only for the cases in which the first sampling did not yield enough DNA and the DNA extraction of the second replicate was added to the first replicate. Libraries were sequenced on the NovaSeq-6000 platform (2 × 150 bp reads). Shotgun metagenomics generated an initial set of 361 samples.

### Sequence preprocessing and taxonomic/functional potential profiling

The generated raw metagenomes were processed with Trim Galore (v. 0.6.6) with the following parameters: “--nextera --stringency 5 --length 75 --nextseq 20 --max_n 2 --trim-n --dont_gzip --no_report_file --suppress_warn”. Human and bacteriophage phiX174 DNA (Illumina spike-in) was then removed using BowTie2^23^ (v. 2.3.4.3) by mapping the reads against the corresponding reference genomes. We used MetaPhlAn^24^ (v. 4) for the taxonomic characterization of the sampled microbial community and by setting “--stat_q 0.2”. This is an improved version of MetaPhlAn (v. 3) having as major changes the delineation of taxa in terms of SGBs using the clustering-based approach proposed in ref. 25, and the use of a much larger marker database derived from both isolate genomes and metagenome-assembled genomes (MAGs). Finally, functional profiling was performed through HUMAnN^24^ (v. 3.0.0.).

From the 361 sequenced samples, we excluded 25 samples with a low number of reads (i.e., <100,000 reads after pre-processing) and 16 samples having possible contamination issues (i.e., having an estimated relative abundance of *Cutibacterium acnes* >10% in MetaPhlAn profiles). This resulted in the final set of 320 samples coming from 91 subjects (for a total of 11 G of reads), which contributed as follows: 43 patients (H:15, M:11, P:17) contributing with both an implant and a contralateral site, 45 patients (H:14, M:17, P:14) contributing with an implant but not with a contralateral site, and 3 patients (H:2, M:1, P:0) contributing with only the contralateral site (Supplementary Data S2).

### Statistical analysis

Principal Components Analysis (PCA; Fig. 1a) was built on taxonomic profiles via custom python scripts based on Scipy (v. 1.7.3) and Scikit-learn (v. 1.0.2)^26,27^ libraries. PERMANOVA was performed using the Scikit-bio python library (v. 0.5.6; 1000 permutations). We computed the probability density function (PDF) of the points resulting from PCA using the gaussian_kde function implemented in Scipy (v. 1.7.3). This was done for the samples of each study group (i.e., peri-implantitis, mucositis, and healthy) independently. Then, for each point, we extracted the values from the peri-implantitis and the healthy groups. For each sample, density values represented distances from the center of the two groups; this means that high values were associated with samples close to the center. Then, we computed the ratio between peri-implantitis and healthy density values which estimates the similarity of the microbiome profile to the health or peri-implantitis microbiome. The resulting plots represented such ratios in function of the study condition for taxonomic (Fig. 1b) and functional (Fig. 1c) profiles.

We performed biomarker discovery using LEfSe^28^ (v. 1.1.01) on MetaPhlAn taxonomic abundance profiles and on HUMAnN gene family relative abundance profiles. More specifically, the most relevant species reported in Fig. 2 were identified by comparing healthy and peri-implantitis samples (only at T0 and excluding contralateral samples) with LEfSe and by considering the top-10 features based on the effect size (*P* < 0.05). Analyses involving multiple hypothesis testing corrections were done using the Benjamini–Hochberg approach implemented in the Python library Statsmodel (v. 0.13.1).

### Reporting summary

Further information on research design is available in the Nature Research Reporting Summary linked to this article.

### Supplementary information

Supplementary Material

Supplementary Data S1

Supplementary Data S2

Reporting Summary

## Data Availability

All metagenomes have been deposited and are available at the NCBI Sequence Read Archive under accession BioProject PRJNA547717.
