# Supplementary material for: Favorable subgingival plaque microbiome shifts are associated with clinical treatment for peri-implant diseases
Source: NPJ Biofilms Microbiomes. 2024 Feb 19;10:12. doi: 10.1038/s41522-024-00482-z (PMC10876967; doi:10.1038/s41522-024-00482-z)
Supplement: Supplementary file 1 — Supplementary Material [file 41522_2024_482_MOESM1_ESM.pdf]

Favorable subgingival plaque microbiome shifts are associated with clinical treatment for peri-implant diseases

Davide Bazzani [1], Vitor Heidrich [2], Paolo Manghi [2], Aitor Blanco-Miguez [2], Francesco Asnicar [2], Federica Armanini [2], Sara Cavaliere [3], Alberto Bertelle [4], Federico Dell'Acqua [4], Ester Dellasega [4], Romina Waldner [4], Daniela Vicentini [4], Mattia Bolzan [1], Cristiano Tomasi [1,5], Nicola Segata [2,*], Edoardo Pasolli [3,*], Paolo Ghensi [1,2*]

1. PreBiomics S.r.l., Trento, Italy
2. Department CIBIO, University of Trento, Trento, Italy
3. Department of Agricultural Sciences, University of Naples Federico II, Portici, Italy
4. Private Practice, Trentino-Alto Adige, Italy
5. Department of Periodontology, Institute of Odontology, Sahlgrenska Academy, University of Gothenburg, Gothenburg, Sweden.

* Correspondence: paolo.ghensi@prebiomics.com; edoardo.pasolli@unina.it; nicola.segata@unitn.it

Supplementary Material

Supplementary Figures

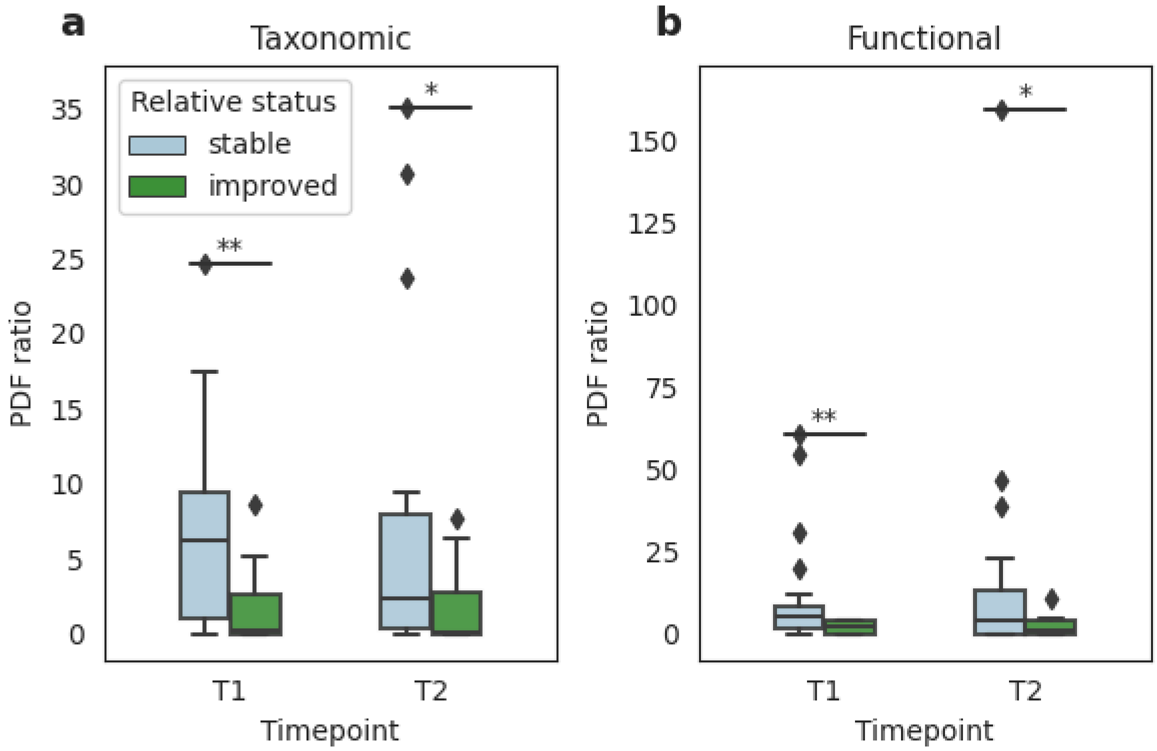

Supplementary Fig. 1: The plaque microbiome shift toward a healthier composition after treatment is greater in individuals with improved clinical condition. Probability density functions (PDFs) computed on diseased samples and normalized on the healthy ones for both **a**) taxonomic and **b**) functional data (**Methods;** p-values computed using Wilcoxon-Mann-Whitney test) were used to assess proximity to the healthy state. Initially diseased individuals were classified as 'stable' or 'improved' according to the clinical status of the implant at T1 or T2 in relation to T0 (**Methods**). Whiskers are ±S.D.

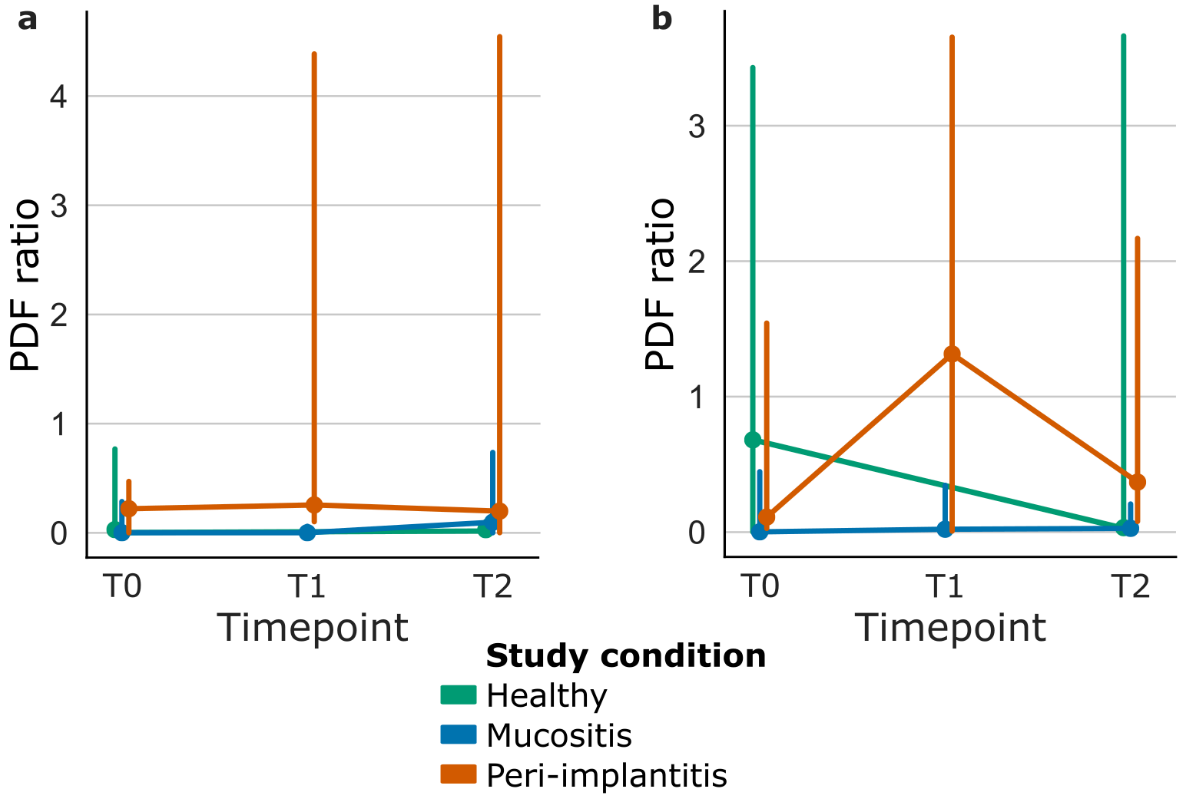

Supplementary Fig. 2: The plaque microbiome in contralateral sites did not change in time even after intervention. The same type of plot depicted in **Fig. 1** for dental implants is shown here for the microbiome associated with contralateral sites. Density plot variations computed on **a**) taxonomic and **b**) functional data (**Methods**) showed no statistically significant changes in the healthy contralateral site independently from the disease status of the subject (i.e., healthy, affected by mucositis, and affected by peri-implantitis).

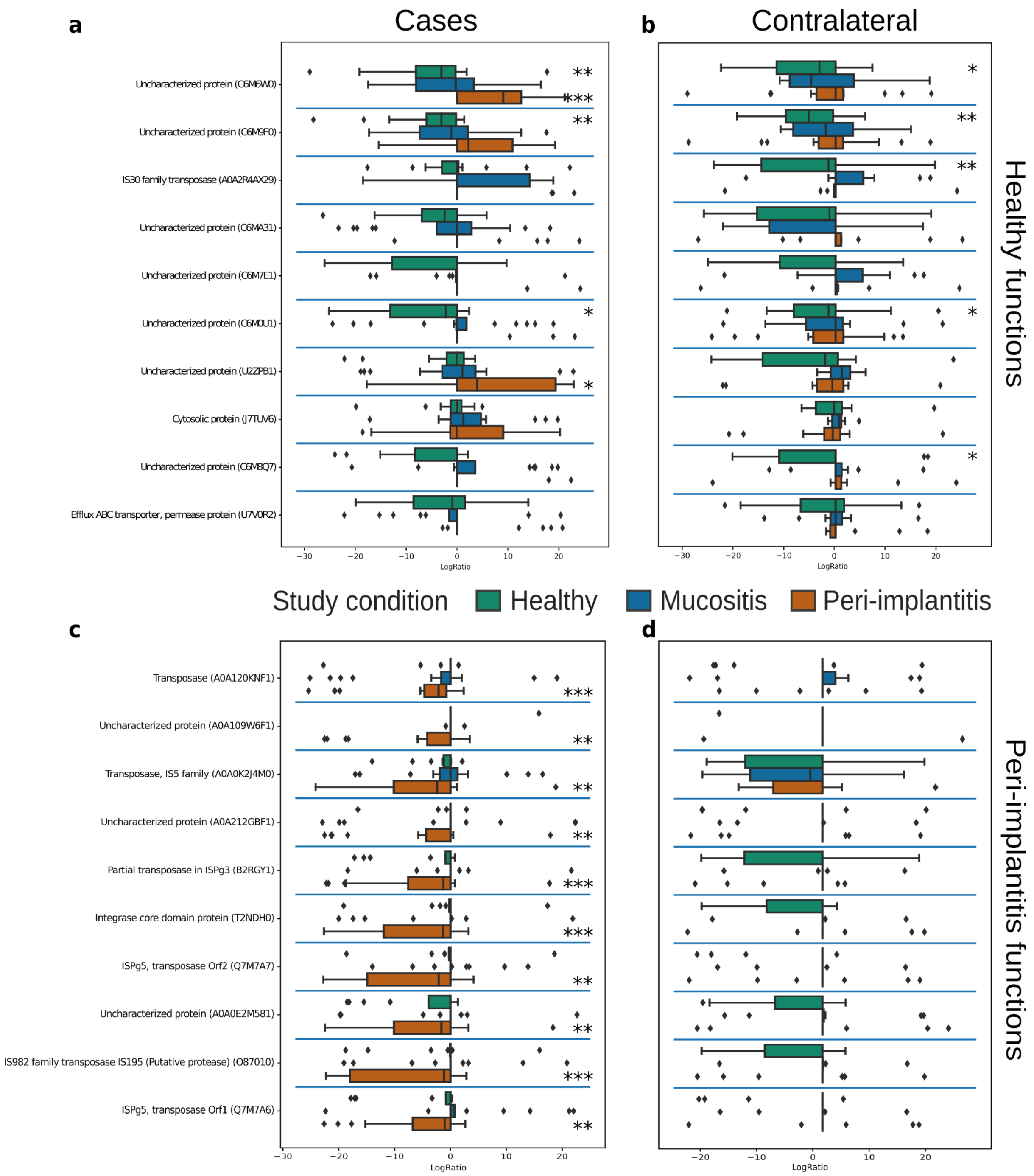

Supplementary Fig. 3: The plaque microbiome functional potential changed in peri-implantitis sites after intervention. We report changes in gene family relative abundances as the (base 2 logarithm) ratio of the abundances at T2 and T0. This logRatio is reported for (a, c) case and (b, d) contralateral samples and stratified by study condition. Contralateral samples were grouped according to the diagnosis of the respective case implant. We report the top-10 UniRef90 gene families identified as the ones most enriched in (a, b) health and (c, d) peri-implantitis through LEfSe (**Methods**). p-value: * < 0.1; ** < 0.05; *** < 0.01. Whiskers are ±S.D.

Supplementary Data

Supplementary Data S1: Summary of the 320 metagenomes stratified by sampling site (i.e., implant, contralateral_teeth, and contralateral_implant), study condition (i.e., healthy, mucositis, and peri-implantitis), and time point (i.e., T0, T1, and T2).

Supplementary Data S2: Metadata information for the 320 analyzed metagenomes.
