# Supplementary material for: Favorable subgingival plaque microbiome shifts are associated with clinical treatment for peri-implant diseases
Source: NPJ Biofilms Microbiomes. 2024 Feb 19;10:12. doi: 10.1038/s41522-024-00482-z (PMC10876967; doi:10.1038/s41522-024-00482-z)
Supplement: Supplementary file 4 — Reporting Summary [file 41522_2024_482_MOESM4_ESM.pdf]

nature portfolio

Corresponding author(s): Ghensi Paoli, Pasolli Edoardo, Segata Nicola

Last updated by author(s): Jan 23, 2024

Reporting Summary

Nature Portfolio wishes to improve the reproducibility of the work that we publish. This form provides structure for consistency and transparency in reporting. For further information on Nature Portfolio policies, see our Editorial Policies and the Editorial Policy Checklist.

Statistics

For all statistical analyses, confirm that the following items are present in the figure legend, table legend, main text, or Methods section.

n/a	Confirmed	
☐	☒	The exact sample size (n) for each experimental group/condition, given as a discrete number and unit of measurement
☐	☒	A statement on whether measurements were taken from distinct samples or whether the same sample was measured repeatedly
☐	☒	The statistical test(s) used AND whether they are one- or two-sided<br>*Only common tests should be described solely by name; describe more complex techniques in the Methods section.*
☐	☒	A description of all covariates tested
☐	☒	A description of any assumptions or corrections, such as tests of normality and adjustment for multiple comparisons
☐	☒	A full description of the statistical parameters including central tendency (e.g. means) or other basic estimates (e.g. regression coefficient) AND variation (e.g. standard deviation) or associated estimates of uncertainty (e.g. confidence intervals)
☐	☒	For null hypothesis testing, the test statistic (e.g. F, t, r) with confidence intervals, effect sizes, degrees of freedom and P value noted<br>*Give P values as exact values whenever suitable.*
☒	☐	For Bayesian analysis, information on the choice of priors and Markov chain Monte Carlo settings
☒	☐	For hierarchical and complex designs, identification of the appropriate level for tests and full reporting of outcomes
☐	☒	Estimates of effect sizes (e.g. Cohen's d, Pearson's r), indicating how they were calculated

Our web collection on statistics for biologists contains articles on many of the points above.

Software and code

Policy information about availability of computer code

Data collection	No software was used for microbiome data collection.
Data analysis	Trim Galore v. 0.6.6; BowTie2 v. 2.3.4.3; MetaPhlAn v. 4; HUMAnN v. 3.0.0; Scipy v. 1.7.3; Scikit-learn v. 1.0.2; Scikit-bio v. 0.5.6; LEfSe 28 ; v. 1.1.01; Statsmodel v. 0.13.1; custom python scripts

For manuscripts utilizing custom algorithms or software that are central to the research but not yet described in published literature, software must be made available to editors and reviewers. We strongly encourage code deposition in a community repository (e.g. GitHub). See the Nature Portfolio guidelines for submitting code & software for further information.

Data

Policy information about availability of data

All manuscripts must include a data availability statement. This statement should provide the following information, where applicable:

- Accession codes, unique identifiers, or web links for publicly available datasets
- A description of any restrictions on data availability
- For clinical datasets or third party data, please ensure that the statement adheres to our policy

All metagenomes have been deposited and are available at the NCBI Sequence Read Archive under accession BioProject PRJNA547717.

Research involving human participants, their data, or biological material

Policy information about studies with human participants or human data. See also policy information about sex, gender (identity/presentation), and sexual orientation and race, ethnicity and racism.

Reporting on sex and gender	Gender was considered in this study and we had 51 males and 51 females.
Reporting on race, ethnicity, or other socially relevant groupings	Not relevant for our study.
Population characteristics	We enrolled for this study 102 patients (51 males, 51 females; mean age 62.54±9.85 years) from three categories: healthy (H: 40), mucositis (M: 29), and peri-implantitis (P: 33). Each patient contributed with one implant in addition to the healthy contralateral site.
Recruitment	Both male and female patients, having dental implants and a regular maintenance of their dental implants were recruited from six private practices in the Province of Trento (Italy). The study protocol was explained to each subject and a signed informed consent was obtained. Inclusion criteria included: good general health as evidenced by the medical history; being at least 18 years old; not having fewer than 8 teeth; having at least one functioning oral implant restored with crowns or prostheses for at least 1 year. Exclusion criteria included: pregnancy or lactation; human immunodeficiency virus; use of immunosuppressant medications, bisphosphonates, or steroids; use of chlorhexidine mouthwash or gel during the previous 2 weeks; oral prophylactic procedures within the preceding 3 months; intake of systemic antibiotics or probiotics within the past 6 months.
Ethics oversight	This study was approved by the ethics committee of the University of Trento (no. 2015-024) and was conducted in accordance with the guidelines of the World Medical Association Declaration of Helsinki.

Note that full information on the approval of the study protocol must also be provided in the manuscript.

Field-specific reporting

Please select the one below that is the best fit for your research. If you are not sure, read the appropriate sections before making your selection.

☒ Life sciences ☐ Behavioural & social sciences ☐ Ecological, evolutionary & environmental sciences

For a reference copy of the document with all sections, see nature.com/documents/nr-reporting-summary-flat.pdf

Life sciences study design

All studies must disclose on these points even when the disclosure is negative.

Sample size	Sample size was computed for the original cross-sectional study (Ghensi et al, npj Biofilms and Microbiomes, 2020). As the current study leverages on this previously analyzed cohort to evaluate longitudinal changes, no sample size calculation specific for this study was performed.
Data exclusions	Eleven patients were excluded due to failure in DNA extraction or library preparation for sequencing.
Replication	Not relevant for our study.
Randomization	Patients were divided into three groups based on the health status of the implant: (a) healthy (H, patients with at least one healthy implant and no implants with mucositis or peri-implantitis), (b) mucositis (M, patients with at least one implant with mucositis and no implants with peri-implantitis), and (c) peri-implantitis (P, patients with at least one implant with peri-implantitis).
Blinding	Not relevant for our study.

Reporting for specific materials, systems and methods

We require information from authors about some types of materials, experimental systems and methods used in many studies. Here, indicate whether each material, system or method listed is relevant to your study. If you are not sure if a list item applies to your research, read the appropriate section before selecting a response.

Materials & experimental systems

n/a	Involved in the study
☒	☐ Antibodies
☒	☐ Eukaryotic cell lines
☒	☐ Palaeontology and archaeology
☒	☐ Animals and other organisms
☒	☐ Clinical data
☒	☐ Dual use research of concern
☒	☐ Plants

Methods

n/a	Involved in the study
☒	☐ ChIP-seq
☒	☐ Flow cytometry
☒	☐ MRI-based neuroimaging

Plants

Seed stocks

Report on the source of all seed stocks or other plant material used. If applicable, state the seed stock centre and catalogue number. If plant specimens were collected from the field, describe the collection location, date and sampling procedures.

Novel plant genotypes

Describe the methods by which all novel plant genotypes were produced. This includes those generated by transgenic approaches, gene editing, chemical/radiation-based mutagenesis and hybridization. For transgenic lines, describe the transformation method, the number of independent lines analyzed and the generation upon which experiments were performed. For gene-edited lines, describe the editor used, the endogenous sequence targeted for editing, the targeting guide RNA sequence (if applicable) and how the editor was applied.

Authentication

Describe any authentication procedures for each seed stock used or novel genotype generated. Describe any experiments used to assess the effect of a mutation and, where applicable, how potential secondary effects (e.g. second site T-DNA insertions, mosiacism, off-target gene editing) were examined.
